# Surgical treatment of severe thoracic kyphosis and neurological deficit in a patient with Gorham–Stout syndrome: A case report and literature review

**DOI:** 10.3389/fsurg.2022.981025

**Published:** 2022-08-08

**Authors:** Hanwen Zhang, Chaofan Han, Daming Pang, Hai Yong, Jincai Yang, Peng Yin, Lijin Zhou

**Affiliations:** Beijing Chaoyang Hospital, Capital Medical University, Beijing, China

**Keywords:** gorham-Stout syndrome, halo-pelvic traction, VCRs osteotomy, chylothorax thoracic kyphosis, spine deformity

## Abstract

**Background:**

Gorham–Stout syndrome is an uncommon condition with a varied clinical presentation and unclear cause that is characterised by a proliferation of lymphatic capillaries and severe regional osteolysis. Spinal and visceral involvement increases the syndrome's morbidity and mortality rates. Here, we report about a male patient with Gorham's disease who developed local kyphosis and neurological disorders due to massive osteolysis.

**Case presentation:**

A 13-year-old male patient presented with progressive kyphosis and massive osteolysis of the thoracic vertebrae. Halo-pelvic traction and vertebral column resection osteotomy were performed to reconstruct the spine and prevent disease progression. The entire lesion was resected, and an artificial vertebra filled with allograft bone was used to achieve temporary stability. Although the patient presented with chylothorax following surgery, which required thoracic drainage, the patient did achieve a satisfying outcome.

**Conclusions:**

Limited by the number of GSS cases with spinal involvement and chylothorax manifestations, halo-pelvic distraction as a preoperative preparation and vertebral column resection osteotomy provide a novel avenue for managing this disease.

## Introduction

Jackson et al. initially described Gorham-Stout syndrome (GSS), also known as phantom bone disease or vanishing bone disease, in 1883 (1). However, it was not considered an independent disease until Gorham and Stout described it in 1955 (2). GSS is a rare disorder, with fewer than 300 reported cases. It is characterised by the proliferation of endothelial-lined vessels in bone and massive type IV osteolysis, described by Hardegger et al. (3). The syndrome has no sex or age predilection, although it most frequently presents in children and adolescents. No genetic predisposition has been established (4). The disease's related progressive resorption of bone results in severe physical deformities, impairments, and potentially fatal complications (5). Unfortunately, the underlying aetiology of GSS is still unknown, leaving patients with the condition with few treatment choices. Chemotherapy, radiation therapy, bisphosphonates, calcium supplements, interferon, vitamins, calcitonin, and embolization are among the non-surgical therapeutic options (6, 7). Patients who have neurological deficit or deformity have been advised to undergo surgery. However, it hasn't been demonstrated that any one type of treatment is more effective than another.

Thus, this study reported the case of a 13-year-old male teenager with GSS who developed local kyphosis and neurological disorders due to massive osteolysis of the left 8–11 ribs, T10, T11, bilateral ilium, left femoral head, and the upper end of the right femur.

## Case presentation

**Figure 1:**
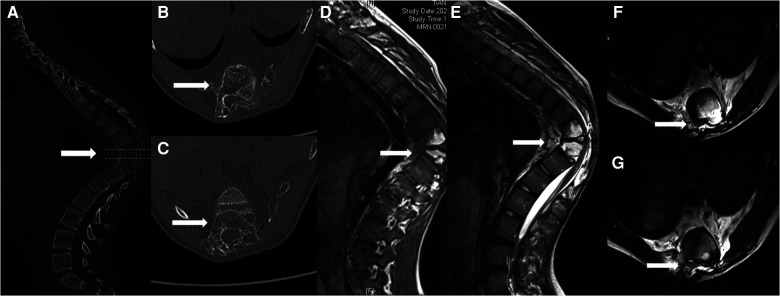
(**A**) Computed tomography images of the thoracic vertebrae indicate marked osteolysis within the T10/T11 vertebrae (arrows); (**B**) Osteolysis within the T10; (**C**) Osteolysis within the T11; (**D**) High-signal intensity was observed on the T1-weighted image within the T10/11 vertebrae (arrows); (**E**) High-signal intensity was observed on the T2-weighted image within the T10/11 vertebrae (arrows); (**F**) spinal cord compression was observed on the T2-weighted image within the T10 level (arrows); (**G**) spinal cord compression was observed on the T2-weighted image within the T11 level (arrows).

Three years ago, the patient observed a noticeable bulging of his back but no trunk tilt, back pain, or limp. Conservative treatment was provided by another hospital. However, the thoracic kyphosis continuously progressed, and the patient presented with myelopathy symptoms, such as increased muscle tone (American Spinal Injury Association [ASIA] category D). Three-dimensional whole-spine computed tomography (CT) showed a low-density shadow on the T10/11 vertebral body and pedicle, involving the left 8–11 ribs, indicating the possibility of a benign bone tumour (Figure 1). Magnetic resonance imaging (MRI) also demonstrated hyperintense signals on both T1WI and T2WI of the T10/11 vertebral body (Figure 1). Positron emission tomography (PET)-CT demonstrated decreased bone density of the right parietal bone, left 8–11 posterior ribs, T10, T11, bilateral ilium, left femoral head, and right upper femur, but no active metabolism. An incisional biopsy was performed to obtain a 3-cm rib specimen and abnormal soft tissue of the paravertebral body, the histopathology of the latter showed no obvious abnormality. Halo-pelvic traction was performed to delay the neurological progression and gain time to ensure an accurate diagnosis.

**Figure 2:**
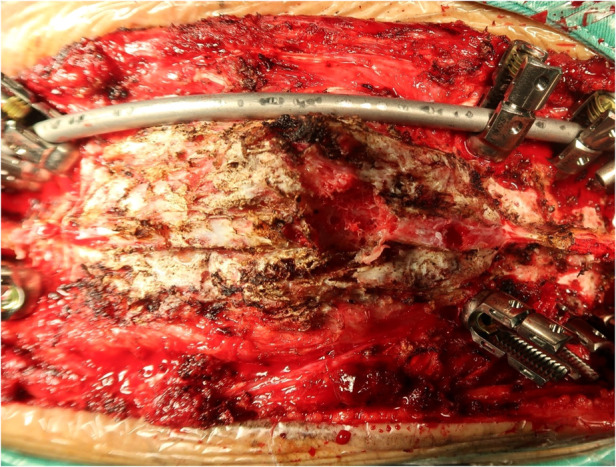
Surgical field of the spine, which revealed thin cortical bone, sparse cancellous bone, and porous cancellous surface that appeared highly soft and spongy.

**Figure 3:**
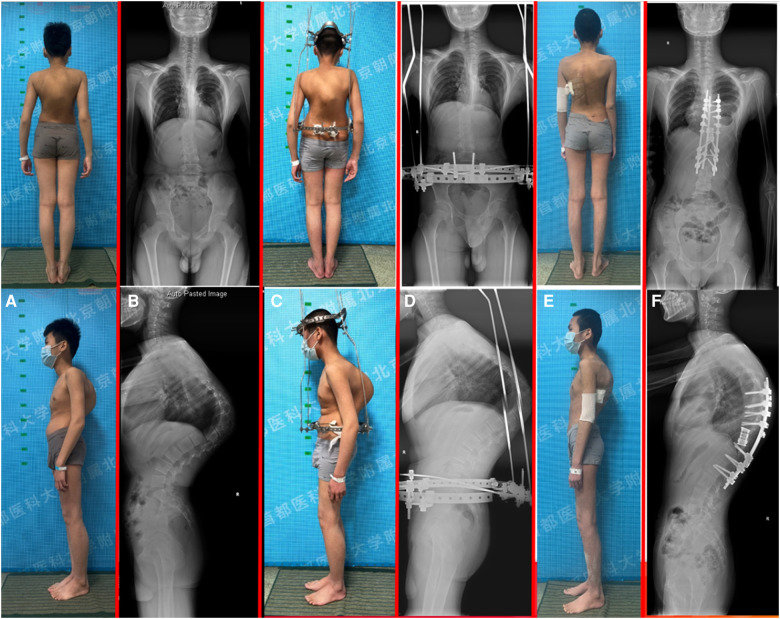
A 13-year-old male with progressive kyphosis and massive osteolysis in the thoracic vertebrae. (**A**) patient's preoperative appearance; (**B**) preoperative standing whole-spine radiograph demonstrated a thoracic kyphosis curve of 122°; (**C**) patient's halo-pelvic traction appearance; (**D**) standing whole-spine radiograph after halo-pelvic traction; (**E**) patient's postoperative appearance; (**F**) postoperative standing whole-spine radiograph demonstrated the thoracic kyphosis was reduced to 48°, with a correction rate of 60.8%.

**Figure 4:**
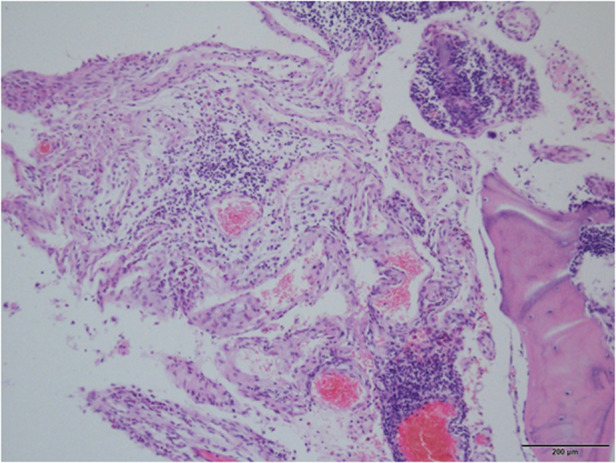
The proliferating fibrous tissues and numerous thin-walled blood vessels in the bone cavity are visible in histological pictures of the removed thoracic vertebrae, but there is no sign of cellular atypia or osteogenesis reactions.

After three months of halo-pelvic traction, the patient developed myelopathy which manifested byincreased muscle tone and tendon hyperreflexia in the lower extremities (ASIA D). A posterior spinal fusion with T10-T11 vertebral column resection (VCR) osteotomy and artificial vertebral body replacement was performed to decompress the spinal cord and reconstruct the spine. During the surgery, lightly hooking the lamina with a nerve peeler destroyed the lamina bone, showing osteoporosis, thin cortical bone, sparse cancellous bone, and a porous cancellous surface, which appeared very soft and spongy (Figure 2). Post-surgery, the patient experienced neurological improvement, regaining normal muscular strength in the lower extremities (ASIA E) (Figure 3). Histological examination of the resected T10-T11 vertebral body and lamina revealed broken mature bone and cartilage, a trabecular bone section that was sparse and thin, along with proliferation of lymph and blood vessels between the trabeculae (Figure 4). The diagnosis of GSS was made based on radiographic, clinical, and pathological findings. Medical treatment was initiated by oral administration of alendronate sodium hydrate (35 mg per week), calcium carbonate, and vitamin D3 tablets.

A week after surgery, the patient complained of troubled breathing. CT revealed bilateral pleural effusion, more pronounced on the left side, resulting in secondary left pulmonary insufficiency. However, none of the pedicle screws were misplaced. Thoracentesis tube drainage was performed under ultrasound guidance. As the resulting drainage was chyle-like, the patient was diagnosed with chylothorax. Surgical thoracic duct ligation was performed, and total parenteral nutrition (TPN) was administered, which seemed effective initially, but symptoms recurred following the initiation of a fat-free diet. The drainage decreased with additional tube drainage, TPN, and strict fasting, which relieved his dyspnoea. After twomonths of fasting and TPN, chylothorax was contained. At the twelve-month follow-up visit at the local hospital, the patient had nearly achieved complete remission without any complication.

## Discussion and conclusions

Spinal GSS is frequently misdiagnosed or overlooked when spinal deformity is originally diagnosed due to ignorance of this rare disease. The diagnosis of GSS is based on radiological, clinical and histological findings after excluding tumour, metastasis, or infection. Plain x-ray show osteolysis or pathological fractures. CT and MRI are useful for evaluating disease extension and soft tissue involvement. CT imaging of patients with GSS shows multilevel generalised osteolytic lesions, with unclear fluid attenuation close to the osseous changes. In contrast, MRI demonstrates hyperintense signals on both T1WI and T2WI, while the unaffected segments show normal signal intensity (8), although considerable variability is recognised. The heterogeneous signal is attributed to the presence of ﬁbrous tissue within the lesions, but once again, considerable variability exists (9–11). In our case, whole-spine three-dimensional CT and MRI examinations revealed iconic GSS findings.

Although the clavicle, scapula, humerus, ribs, and pelvis are most frequently affected, GSS can affect any bone in the body. Chang-Zhi et al. reported 11 GSS patients with spinal deformity. They believed that spinal anomalies, particularly structural kyphosis, rapidly arise as a result of subcortical osteolytic lesions in asymmetrical vertebral locations. Additionally, a severe pathological fracture and an intra-spinal haematoma can compress the spinal cord and nerves, resulting in myelopathy and weakening of the paravertebral muscles (8, 12–14).

Gorham's disease is insidious in origin. Patients are often asymptomatic because the bone erosion itself is painless; however, a pathological fracture often leads to the discovery of the disease. According to a recent clinical case series by Rana and Buonuomo et al, delay in diagnosis after first symptoms played a crucial role in determining the rate of morbidity (15). While known to cause deformity, GSS may be life-threatening when it involves the viscera or chest, resulting in chylothorax. Moreover, although extremely rare, involvement of the spine is associated with high mortality and morbidity.

Due to the lack of knowledge regarding the aetiology of Gorham's disease, no established standard treatment exists. Radiation therapy, chemotherapy, anti-resorptive medications (bisphosphonates), and hemangiomatosis therapy help compensate medical care (8). The osteolytic lesions can be removed and rebuilt surgically, and the fracture can also be treated. Radiation therapy, braces, or halo traction are all options for treating spinal lesions. In patients with spinal involvement that have undergone surgical treatment, instability and dislocation brought on by extensive bone loss are common findings that need to be treated right away. Due to technical difficulties, graft resorption, and other causes, spinal cord decompression and column stabilization therapy is troublesome (16, 17). For individuals with severe deformity, several levels of spinal involvement, or neurologic deficiency, surgical intervention may be necessary. This may involve rigid fixation with instrumentation, decompression, or spondylectomy.

We believe that GSS spinal lesions require complete resection to prevent further progression. However, posterior-only treatments using posterior column osteotomies or anterior/posterior operations combined with acute correction are frequently associated with increased perioperative morbidities. In our case, the patient demonstrated progressive focal kyphosis and neurologic deficit. Neurological symptoms occur following spinal cord compression, which conservative treatment cannot resolve. Prior to corrective operations, halo-pelvic distraction can lessen the severity of the kyphotic deformity, making the final surgery simple and easing neurological symptoms.

In GSS, until they exhibit clinical symptoms such pathologic bone fracture, subcutaneous swelling, or even pericardial effusion or chylothorax, which is the abnormal buildup of chyle in the region between the lungs and chest cavity, these alterations may go undiagnosed (18, 19).

Chylothorax is a common and severe complication of GSS, occurring in approximately 25% of patients (5) that usually presents with thoracic vertebral bony osteolysis (20). Chylothorax in GSS patients is presumed to originate from either an invasion of pre-existing bone lesions into the pleural cavity or a direct invasion of lymphatic dysplasia into the thoracic duct (20). Patients with chylothorax may have respiratory failure and typically have a worse prognosis since this effusion may recur, necessitating recurrent thoracentesis. About 43.6% of patients with chylothorax have reported fatal outcomes, which is much higher than that of patients without it (18). Although there is no standard solution for patients with GSS and chylothorax, tube drainage, TPN, and strict fasting are used therapeutically. Although our surgical procedure (thoracic duct ligation) originally appeared to be beneficial, more time is required to establish whether there has been a renewed resorption of bone or if it has progressed.

In conclusion, surgical removal of the entire lesions, solid fusion, and realignment of the spine are necessary for treating patients with GSS involving the spine, especially with neurological symptoms or progressive spinal deformity. Limited by the number of GSS cases with spinal involvement and chylothorax manifestations, our treatment choice provides a novel avenue for managing this disease.

Thoracic kyphosis, Spine deformity

## Data Availability

The raw data supporting the conclusions of this article will be made available by the authors, without undue reservation.
